# Some Current Topics

**Published:** 1909-01-16

**Authors:** 


### Motoring Notes.

### SOME CURRENT TOPICS.

### One or Two Cylinders.

It may have been noticed in the description of the small cars suitable for medical men which has been given in previous articles that the two-cylinder engine largely predominates. It must not, however, be assumed from this that I personally favour this type as compared with the single-cylinder variety. On the contrary, I may say that for the owner who more or less looks after his car himself, as a great many doctors do, I consider that a really good single-cylinder engine has many points in its favour. There are numerous people who have the impression that a small one-cylinder car cannot in any way be considered comfortable, and that it is always noisy and full of vibration. That such was the case some years ago cannot be denied, but at the present time there are many single-cylinder cars whose running cannot be distinguished from that of two or four-cylinder vehicles. Whilst writing, a particular instance occurs to me which very forcibly convinced me of the great improvement made by manufacturers during the last year or so in regard to the silence of modern single-cylinder engines.

### Silent Single-cylinder Cars.

I was in a garage one day preparatory to driving home, and had to wait for a car in front of mine to go out. The driver of the car started his engine, which, from the size of the bonnet, could not be of the four-cylinder type. The engine was running so quietly that I thought it a remarkably good two, and on telling him so was astounded to learn it was only a single-cylinder. It was, however, a single-cylinder car of the very highest class, the name of which perhaps it would be invidious to mention, but which I shall be pleased to give to any reader, and the point that struck me so forcibly was the fact of the engine being so quiet when running free in a garage, a totally different thing from quietness on the road.

Noise, after all, is only a matter of correct design of engine, gear, and silencer, although the beat of a single-cylinder engine being marked obtrudes itself more on one's notice. Some of my readers may remember that a gentleman whose name is just now well known in the motor world in connection with a new engine a short time ago remarked that what the six-cylinder people called silence was merely a continuity of noise.

There is naturally more vibration in the single-cylinder car than in the large multi-cylinder type, but this is, to a great extent, due to the fact that the car is light in weight. Lightness, however, has its compensations in regard to wear on tyres and consumption of petrol, both very important points worthy of consideration by the medical motorist.

### Precautions in Frosty Weather.

Just now and for some time onwards, as the experience of the past tells us, we may expect the most severe weather of the year, and it behoves every car owner to take all precautions against the freezing of the water in his radiators, which invariably results in a cracked cylinder, owing to expansion of the water-jacket. There are various ways by which this can be avoided. Some people go so far as to run the water off every evening before putting the car away. This takes a little time, and necessitates refilling the water system next morning before taking the car out. For those who only use the car occasionally this may be a very excellent way out of the difficulty; but for the medical man who uses his car daily, not to speak of at night time, when a car is usually wanted to be ready at once, it is not only troublesome, but causes considerable delay in starting off. Another way is to keep the motor house sufficiently heated. I fancy there are very few doctors' carriage houses in which there is a proper system of heating by hot air or hot water, for this alone is safe where petrol is concerned. In this connection, however, several stoves have recently been put on the market which burn a patent fuel, and are considered safe to use in a motor house. These I intend to describe another time. Failing this second method we come to the third, which is the one most generally followed, and which I think it wise to adopt even when there is some system of heating the motor house, since a car has at times of severe frost to stand out of doors for periods quite sufficiently long to freeze the water. This is the use of anti-freezing solutions. These mainly consist of solutions of glycerine, calcium chloride, and wood alcohol, in varying strengths.

### Anti-freezing Solutions.

Solutions of glycerine are favoured by many, as they are fairly cheap; it is not necessary to use the best, and ordinary crude brown glycerine at about sixpence a pound will quite meet the case. A 20 per cent. solution will stand about 7° of frost, and a 25 per cent. solution about 12°. These are the strengths most generally used, although in extremely severe weather it might be advisable to use still stronger mixtures. Calcium chloride solutions are also largely employed, although some people consider the solution unsatisfactory owing to the possible deposition of a film of the salt on the interior of the radiator tubes and of the cylinder body. The solution, however, has a somewhat stronger resistance to frost than glycerine. A 15 per cent. solution will resist 8° of frost, an 18 per cent 14°, and a 25 per cent. solution 26°. The solution is cheap; it must be remembered that calcium chloride is very deliquescent, so that it may be kept well sealed.

Wood alcohol in a 15 per cent. solution has been used with success, as also has methylated spirit; but I have personally no experience of either. There are also several patent preparations advertised for this purpose; but I would strongly advise anyone before using these to satisfy himself that they contain no ingredient having a corrosive action on metals.

"Viator."

